# Substance abuse co-morbidity in schizophrenia: An inpatient study of course and outcome

**DOI:** 10.4103/0019-5545.46072

**Published:** 2005

**Authors:** Tapas K. Aich, Vinod K. Sinha, Christoday R.J. Khess, Shailja Singh

**Affiliations:** * Assistant Professor of Psychiatry, Universal College of Medical Sciences, PB-53, Ranigaon, Bhairahawa, Nepal; ** Assistant Professor of Psychiatry, Central Institute of Psychiatry, Kanke, Ranchi 834006, Jharkhand; *** Professor of Psychiatry, Central Institute of Psychiatry, Kanke, Ranchi 834006, Jharkhand; **** Lecturer in Clinical Psychology, ‘Chetna’—Institute for the Mentally Retarded, Regional Training Centre, Sector-C, Aliganj, Lucknow 226024, Uttar Pradesh

**Keywords:** Schizophrenia, substance abuse, co-morbidity, course and outcome

## Abstract

**Background:**

Differences in opinion exist among researchers in relation to the course and outcome of substance abuse co-morbidity in schizophrenia.

**Aim:**

To compare the pattern of remission of symptoms in positive and negative schizophrenics with and without a history of substance abuse.

**Methods:**

Seventy schizophrenics were divided into two groups based on the history of presence or absence of substance abuse/dependence. Thirty-eight patients (54.3%) were diagnosed as having co-morbid alcohol/substance abuse/dependence. Patients were rated at two-weekly intervals on the Positive and Negative Syndrome Scale (PANSS) with one follow-up rating by the end of the third month. Co-morbid substance abusers were predominantly represented by a positive syndrome and non-abusers by a negative syndrome at the time of admission.

**Results:**

Psychopathology remitted much faster in the substance-abusing group but after discharge, these patients tend to return to their pre-admission state.

**Conclusion:**

The following hypothesis is proposed based on the findings of the present study: ‘Short-term inpatient outcome of substance-abusing schizophrenics is significantly better than non-substance abusing schizophrenics because of the faster rate of remission of their symptoms.’ A corollary to the above hypothesis may be evolved—‘through intense inpatient follow-up one may be able to differentiate substance-abusing and non-abusing schizophrenics’.

## INTRODUCTION

Various types of studies have been carried out to study the relationship between schizophrenia and co-morbid substance abuse. These include self-reports of subjective experiences while using drugs,^1^,^2^ clinical studies^3^–^5^ as well as longitudinal studies.^6^,^7^ However, very few course and outcome studies have been done in this field. Knudsen and Vilmar in a longitudinal study found exacerbation of psychosis among schizophrenic patients who used cannabis.^8^ In a one-year prospective study, Linszen *et al*. found no initial differences in the severity of symptoms according to substance use but, over time, heavy cannabis users showed intermittent increase in psychotic symptoms compared to mild users and non-users.^6^ In a follow-up study of co-morbid inpatients, Rosenthal *et al*. found that, contrary to the majority of schizophrenic patients without substance abuse, the trajectory of symptoms of dual-diagnosis patients changed from predominantly negative to predomi-nantly positive.^9^ Zisook *et al*. concluded that there was no difference between the two groups in impairment and symptomatology.^10^ Cuffel and Chase used 1-year follow-up data of the Epidemiologic Catchment Area (ECA) study and found that individuals who developed active substance use over the year experienced an increase in depression.^11^ Hamera *et al*. found no evidence for increased alcohol or cannabis use following an increase in positive, negative or dysphoric symptoms.^12^ Shaner *et al*. followed schizophrenic patients weekly for 15 weeks and found that cocaine use, assessed by urine tests, was correlated with an increase in the positive symptoms of psychosis.^13^ Kovasznay *et al*. rated their schizophrenic patients twice: during initial take up and at the end of 6 months. A lower (worse) Global Assessment of Functioning (GAF) score and higher (worse) Brief Psychiatric Rating Scale (BPRS) score at 6 months were associated with having a substance abuse disorder for patients with schizophrenia.^7^ Dixon *et al*. did not find any difference between substance-abusing and non-abusing schizophrenics at the time of admission.^14^ However, at discharge, substance-abusing patients showed less severe psychopathology, as measured by total BPRS and Scale for Assessment of Negative Symptoms (SANS) scores.

Thus, differences in opinion exist among researchers in relation to the course and outcome of substance abuse co-morbidity in schizophrenia. Intensive inpatient follow-up studies in these populations are rare. We compared the pattern of remission of symptoms in positive and negative schizo-phrenics with and without a history of substance abuse.

## METHODS

The study was conducted at the Central Institute of Psychiatry, Kanke, Ranchi, India. We interviewed all consecutive male patients with a diagnosis of schizophrenia admitted between 1 March 1998 and 30 September 1998. Of the 88 patients screened, 70 fulfilled the DSM-III-R criteria for schizophrenia and consented to participate; these patients were included in the study.

The patients were divided into two groups based on the presence or absence of alcohol or drug abuse/dependence as defined by the DSM-III-R criteria. Thus, the study population comprised patients with a diagnosis of schizophrenia and co-morbid drug or alcohol abuse/dependence (SAS group). The control population comprised schizophrenic patients with no history of drug or alcohol abuse (NSAS group). Substance/alcohol abusers/dependents were considered as a single group. This simplification is useful for analysis and is based on the international trend in co-morbidity research. Inclusion criteria were male patients with a DSM-III-R diagnosis of schizo-phrenia in the age group of 18–45 years.^15^ Exclusion criteria were evidence of organicity either from the history, clinical examination or laboratory examination, patients who did not consent to participate in the study, and any co-morbid major psychiatric disorder or mental retardation.

The assessment tools used were the Structured Clinical Interview for DSM-III-R (SCID-P)^16^ and Positive and Negative Syndrome Scale (PANSS).^17^ SCID is a semi-structured interview schedule for diagnosing major axis-I and axis-II disorders. PANSS was developed as a more rigorously operationalized method for evaluating positive, negative and other symptom dimensions in schizophrenia. A series of studies provided enough evidence of suitable psychometric properties of PANSS for typological and dimensional assessment of distinct syndromes in schizophrenia.^17^ Good to excellent reliability was found on all scale scores and most items of PANSS.^18^

### Assessment technique

At the time of admission, we interviewed the patients' relatives to gather information about the index episode, as well as about previous episodes of psychiatric illnesses, if any. Information regarding the history of substance abuse or dependence was also gathered from relatives and close friends. Relevant sociodemographic, illness and treatment variables were recorded in a specially designed clinical datasheet.

Each of the patients who satisfied the inclusion criteria was interviewed within 48 hours of admission to the hospital. An interview was conducted using SCID-P to confirm the diagnosis of schizophrenia and drug or alcohol abuse/dependence, if any. The severity of psychopathology was assessed by using PANSS. Patients were classified as a positive subtype if they scored 3 or more moderate ratings on the positive scale but less than 3 moderate ratings on the negative scale. Patients were classified as a negative subtype if they exhibited the opposite pattern; i.e. at least 3 moderate ratings on the negative scale but less than 3 moderate ratings on the positive scale. Patients who scored at least 3 moderate ratings on both the scales were regarded as ‘mixed type’. Routine blood and biochemical investigations, and special investigations, as and when needed, were done to rule out the possibility of organic schizophrenic illness.

Subsequent to the primary evaluation, all the patients were followed up for a period of 2 months or till discharge. To assess the rate of recovery and symptomatic outcome, all these patients were rated on PANSS at an interval of 2, 4, 6 and 8 weeks after admission. The patients were reassessed by the end of the third month if they remained in hospital, or called once for a follow-up after discharge from the hospital.

Descriptive statistics and *t* test were performed for the data thus obtained. For detailed statistical analyses, a computer-assisted statistical program (Statistical Program for Social Sciences: SPSS version 7.0) was used.

## RESULTS

Of the total population, 38 (54.3%) had a history of substance abuse/dependence, whereas 32 (45.7%) had no such history. There was no significant statistical difference between the two groups on most demographic and clinical parameters, which made it easier to compare the course of the illness in the two groups (Table 1).

**Table 1:** Comparison between substance-abusing schizophrenics (SAS) and non-substance abusing schizophrenics (NSAS) across various sociodemographic and clinical variables

Variable	SAS (*n*=38)	NSAS (*n*=32)	Total	t/χ^2^ value	p
Age (in years) mean (SD)	31.5 (5.9)	30.5 (7.7)		*t*=0.64	0.5
Education (in years) mean (SD)	8.8 (4.1)	9.6 (4.6)		*t*=0.78	0.4
Socioeconomic status				χ^2^=0.98	0.3
Lower	11	6	17 (24.2)		
Middle and above	27	26	53 (75.8)		
Habitat				χ^2^=4.3	0.04
Rural	26	14	40 (57.1)		
Urban	12	18	30 (42.9)		
Marital status				χ^2^=7.78	<0.01*
Married	29	14	43 (61.4)		
Single	9	18	27 (38.6)		
Occupation				χ^2^=2.53	0.1
Unemployed	13	17	30 (42.9)		
Employed	25	15	40 (57.1)		
Stay in hospital (in days) mean (SD)	41.7 (17.4)	47.5 (19.2)		*t*=−1.3	0.2
Schizophrenia subtypes				χ^2^=6.14	0.2
Paranoid	17	12	29 (41.4)		
Catatonic	2	6	8 (11.4)		
Disorganized	2	5	7 (10.0)		
Undifferentiated	13	7	20 (28.6)		
Residual	4	2	6 (8.6)		

* significant at 0.01 level

Values in parentheses are percentages.

**Fig. 1:**
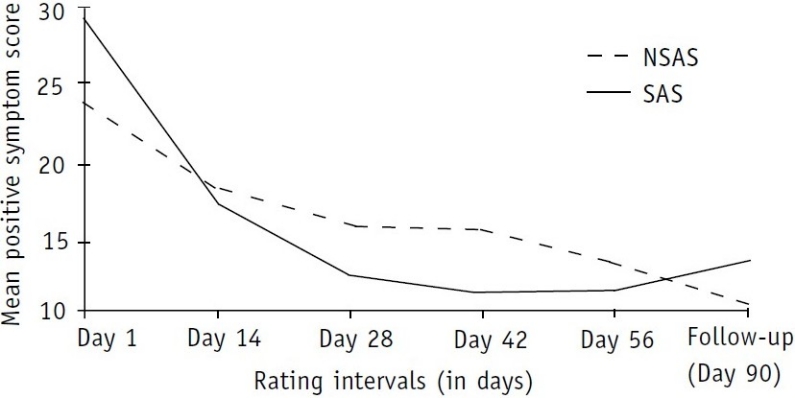
Distribution of positive symptom scores in the two groups NSAS: non-substance abusing schizophrenics; SAS: substance-abusing schizophrenics

An answer to the question ‘Does specific clinical presentation predict the differential outcome in substance-abusing and non-abusing schizophrenics?’ was sought through a series of *t* tests performed in the two groups on a multitude of symptom dimensions recorded at different time intervals (Table 2 and Fig. 1).

**Table 2:** Positive syndrome scores in the two groups

Day	*n*	Mean (SD)	*t*	p
Day 1					
SAS	38	29.4 (8.1)	2.5	0.02*
NSAS	32	23.8 (10.7)		
Day 14					
SAS	36	17.2 (7.4)	−0.8	0.5
NSAS	30	18.5 (7.0)		
Day 28					
SAS	32	12.4 (5.3)	−2.8	<0.01**
NSAS	29	16.2 (5.5)		
Day 42					
SAS	22	11.2 (5.5)	−2.1	0.05*
NSAS	26	15.7 (6.8)		
Day 56					
SAS	10	11.3 (4.2)	−0.8	0.5
NSAS	18	13.7 (7.0)		
Follow-up day					
SAS	18	13.2 (8.8)	0.9	0.3
NSAS	22	10.5 (3.0)		

* significant at 0.05 level;

** significant at 0.01 level

SAS: substance-abusing schizophrenics; NSAS: non-substance abusing schizophrenics

Table 2 shows the distribution of mean positive syndrome scores across the two groups. The mean positive syndrome score on day 1 in the SAS group was significantly higher compared with that of the NSAS group. However, the scoring pattern on subsequent ratings showed that the severity of positive symptoms remitted much faster in the SAS than in the NSAS group. This resulted in a reversal pattern, with the NSAS group scoring significantly higher than the SAS group by the end of weeks 4 and 6. The remission pattern curve showed an upward concavity in the SAS group and a somewhat steady downward slope in the NSAS group. In the two final ratings, there was no significant difference between the two groups but there was another reversal of symptom rating. Follow-up rating at the end of the third month showed that those in the SAS group were steadily regaining their positive symptoms.

**Fig. 2:**
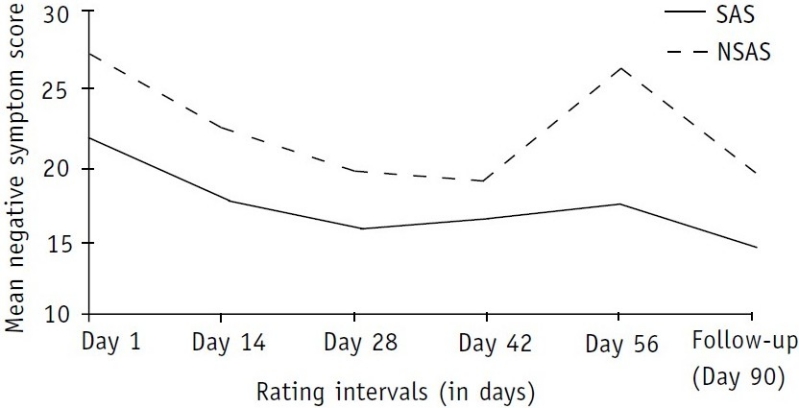
Distribution of negative symptom scores in the two groups SAS: substance-abusing schizophrenics; NSAS: non-substance abusing schizophrenics

A review of the data in Table 3 and Fig. 2 revealed uniformly high negative symptom scoring in the NSAS population in comparison with the SAS. During their inpatient stay, the differences were statistically significant on most ratings.

**Table 3:** Negative symptom scores in the two groups

Day	*n*	Mean (SD)	*t*	p
Day 1				
SAS	38	22.7 (8.9)	−1.9	0.05*
NSAS	32	26.8 (8.8)		
Day 14				
SAS	36	18.1 (7.4)	−2.5	0.01**
NSAS	30	23.0 (8.4)		
Day 28				
SAS	32	15.9 (7.3)	−2.0	0.05*
NSAS	29	19.8 (7.7)		
Day 42				
SAS	25	16.8 (7.7)	−0.7	0.5
NSAS	26	18.9 (8.4)		
Day 56				
SAS	10	17.7 (7.2)	−2.4	0.03*
NSAS	15	26.2 (4.9)		
Follow-up day				
SAS	18	14.8 (4.4)	−1.4	0.2
NSAS	20	19.5 (10.2)		

* significant at 0.05 level;

** significant at 0.01 level

NSAS: non-substance abusing schizophrenics; SAS: substance-abusing schizophrenics

**Fig. 3:**
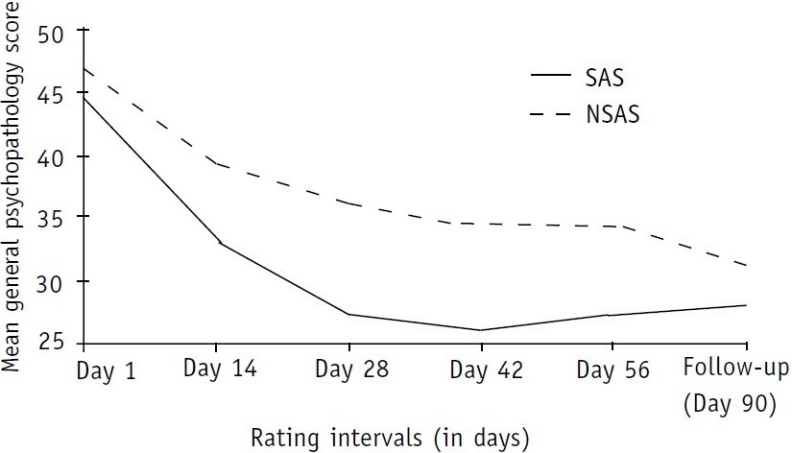
Distribution of general psychopathology scores SAS: substance-abusing schizophrenics; NSAS: non-substance abusing schizophrenics

The results showed persistent increased ratings on general psychopathology scores in the NSAS group in comparison with the SAS group, from day 1 to the day of follow-up (Table 4). Though the difference was not statistically significant on day 1 and the follow-up day, during their inpatient stay, there was a significant difference in all the other ratings between the two groups. Figure 3 shows hyperbolic curves of both the lines, suggesting a steady reduction of symptoms in both the groups, with the SAS group again showing a late surge in symptoms during the follow-up rating.

**Table 4:** General psychopathology scores in the two groups

Day	*n*	Mean (SD)	*t*	p
Day 1					
SAS	38	44.8 (8.4)	−1.1	0.3
NSAS	32	47.4 (11.4)		
Day 14					
SAS	36	33.3 (8.6)	−2.7	<0.01**
NSAS	30	39.4 (9.9)		
Day 28				
SAS	32	27.3 (7.1)	−4.7	<0.01**
NSAS	29	36.2 (7.7)		
Day 42					
SAS	25	26.1 (6.6)	−2.8	<0.01**
NSAS	26	34.6 (9.9)		
Day 56				
SAS	10	27.3 (5.1)	−3.1	0.01**
NSAS	15	34.5 (2.9)		
Follow-up day					
SAS	18	27.9 (9.3)	−0.9	0.4
NSAS	20	31.5 (10.7)		

** significant at 0.01 level

SAS: substance-abusing schizophrenics; NSAS: non-substance abusing schizophrenics

## DISCUSSION

Reports of similar studies are rare in the existing literature. Tables 1–3 and Figs 1–3 show that the severity of psycho-pathology between the two groups varied from time to time during the inpatient stay as well as at the first follow-up at the end of 3 months.

The findings in relation to positive symptom ratings are interesting. It is obvious from Table 2 and Fig. 1 that if an observer decided to rate and report the severity of positive symptoms of these patients at different time points—from their admission to discharge and follow-up—he would be reporting differently each time and one report would contrast and conflict with the other. This contrast and confusion is probably what has been published in the existing literature. Some workers reported significant increase,^19^ some non-significant increase,^20^ some decrease^21^ and some reported significantly lower scores^14^ in positive symptoms in SAS. Sevy *et al*. found no relationship between a history of cocaine abuse (including mixed substance abuse) and global measures of positive, negative and general symptoms using PANSS.^22^ Besides the timing of rating, probable selection bias could also be responsible for such diverse findings. For example, Swofford *et al*.^19^ rated their patients at the time of intake and Drake and Wallach's patient group comprised chronic schizo-phrenics.^23^ Peralta and Cuesta restricted their analysis to cannabis-abusing schizophrenics only.^21^ Dixon *et al*. did not find any difference between the two groups (SAS vs NSAS) at the time of admission.^14^ However, they reported that at the time of discharge, the SAS group had a significantly lower score on positive-symptom BPRS dimensions. Reviewing Table 3 and Fig. 2, we found uniformly low negative symptom scoring in the SAS population in comparison with the NSAS group. A similar scoring pattern was observed in relation to general psychopathology ratings in the two groups (Table 4 and Fig. 3).

The available literature on the subject has several conflicting reports. Dixon *et al*. reported significantly less affective flattening, less anhedonia and less avolition in the SAS group compared with the NSAS group at the time of discharge.^14^ Other studies found that schizophrenics with cocaine abuse had lower levels of negative symptoms than non-abusers.^24^,^25^ Other workers reported no significant difference in negative symptoms between the two groups of schizophrenics.^6^,^7^,^26^–^28^ Kovasznay *et al*., in a 6-month follow-up study, reported that SAS patients had a worse BPRS score, suggesting more psychopathology.^7^ Swofford *et al*. reported a significantly higher severity of illness score on the BPRS among SAS at the onset of their study.^19^,^29^

The present study tried to find out whether SAS and NSAS could be differentiated clinically at the time of (i) admission, (ii) during inpatient stay, (iii) at the time of discharge, and (iv) during follow-up after discharge. The study showed that the two groups differed significantly on both positive and negative symptom ratings at the time of admission. They significantly differed again on negative and general psychopathology ratings at the time of discharge. However, during their first follow-up rating after discharge, these two groups did not differ significantly on any of these three symptom ratings. During their inpatient stay, on all three symptom scales—positive, negative and general psychopathology—the rate of remission of symptoms was faster in the SAS group. These differential rates of remission resulted in significantly higher ratings in the NSAS group across all three domains of psychopathology at the end of their 1-month stay in the hospital. Thus, an intense follow-up of these patients enabled us to differentiate SAS from NSAS.

When the psychopathology ratings on different dimensions on day 56 and the follow-up day were compared, it was found that there was a steady increase in severity ratings on the positive and general psychopathology scale scores, and slowed-down recovery rates on negative and related scales among the substance-abusing population. We may hypothesize that once these patients were discharged from the hospital, they either stopped taking medications or resumed their habit of alcohol/substance abuse. Probably either one or both factors led to an increase in severity of psychopathology with a possibility of relapse of symptoms/illness. That substance abuse is responsible for early relapse has been reported by many researchers.^19^,^30^–^32^ Owen *et al*. speculated about a special high-risk group characterized by substance abuse, non-compliance with medication and lack of outpatient contact among SAS.^31^ These patients report significantly greater symptom severity at 6-month follow-up. The idea that substance abuse is the reason for non-compliance has been challenged by Gupta *et al*.^32^ In a retrospective study, where medication compliance was strictly assured, they reported a significantly higher readmission rate in the substance-abusing population in comparison with non-abusers.

We hypothesize that the short-term inpatient outcome of SAS is significantly better than NSAS because of their faster rate of remission of symptoms. Ries *et al*. also reported that patients with schizophrenia who have a dual diagnosis appear to stabilize faster during acute hospitalization than those without a dual diagnosis.^33^ Contrary to our findings, Ries *et al*. reported that the severity of psychosis was the same at admission for the two groups, though patients with a dual diagnosis were rated as less psychotic at discharge. A corollary to the above hypothesis could be that intense inpatient follow-up may help to differentiate between substance-abusing and non-abusing schizophrenics. This differentiation is important as patients with a dual diagnosis need more intense and collaborative treatment approaches for relapse prevention and a successful long-term outcome.

## LIMITATIONS

The high attrition rate after day 42 made it difficult for us to subsequently make comparative statements between the two groups. However, as the rate of attrition is comparable in both the groups, with an adequate sample for statistical analysis, the results may be accepted for discussion and inference. The results need further replication for wider acceptance among the psychiatric fraternity.
